# DivIVA Interacts with the Cell Wall Hydrolase MltG To Regulate Peptidoglycan Synthesis in Streptococcus suis

**DOI:** 10.1128/spectrum.04750-22

**Published:** 2023-05-22

**Authors:** Qinggen Jiang, Boxi Li, Liangsheng Zhang, Tingting Li, Qiao Hu, Haotian Li, Wenjin Zou, Zhe Hu, Qi Huang, Rui Zhou

**Affiliations:** a State Key Laboratory of Agricultural Microbiology, College of Veterinary Medicine, Huazhong Agricultural University, Wuhan, China; b Cooperative Innovation Center for Sustainable Pig Production, Wuhan, China; c International Research Centre for Animal Diseases (MOST), Wuhan, China; d Institute of Animal Husbandry and Veterinary, Hubei Academy of Agricultural Sciences, Wuhan, China; Centre National de la Recherche Scientifique, Aix-Marseille Université

**Keywords:** DivIVA, MltG, peripheral peptidoglycan synthesis, phosphorylation, *Streptococcus suis*

## Abstract

Bacterial morphology is largely determined by the spatial and temporal regulation of peptidoglycan (PG) biosynthesis. Ovococci possess a unique pattern of PG synthesis different from the well studied Bacillus, and the mechanism of the coordination of PG synthesis remains poorly understood. Several regulatory proteins have been identified to be involved in the regulation of ovococcal morphogenesis, among which DivIVA is an important one to regulate PG synthesis in streptococci, while its mechanism is largely unknown. Here, the zoonotic pathogen Streptococcus suis was used to investigate the regulation of DivIVA on PG synthesis. Fluorescent d-amino acid probing and 3D-structured illumination microscopy found that DivIVA deletion caused abortive peripheral PG synthesis, resulting in a decreased aspect ratio. The phosphorylation-depleted mutant (DivIVA^3A^) cells displayed a longer nascent PG and became longer, whereas the phosphorylation-mimicking mutant (DivIVA^3E^) cells showed a shorter nascent PG and became shorter, suggesting that DivIVA phosphorylation is involved in regulating peripheral PG synthesis. Several DivIVA-interacting proteins were identified, and the interaction was confirmed between DivIVA and MltG, a cell wall hydrolase essential for cell elongation. DivIVA did not affect the PG hydrolysis activity of MltG, while the phosphorylation state of DivIVA affected its interaction with MltG. MltG was mislocalized in the Δ*divIVA* and DivIVA^3E^ cells, and both Δ*mltG* and DivIVA^3E^ cells formed significantly rounder cells, indicating an important role of DivIVA phosphorylation in regulating PG synthesis through MltG. These findings highlight the regulatory mechanism of PG synthesis and morphogenesis of ovococci.

**IMPORTANCE** The peptidoglycan (PG) biosynthesis pathway provides a rich source of novel antimicrobial drug targets. However, bacterial PG synthesis and its regulation is a very complex process involving dozens of proteins. Moreover, unlike the well studied Bacillus, ovococci undergo unusual PG synthesis with unique mechanisms of coordination. DivIVA is an important regulator of PG synthesis in ovococci, while its exact role in regulating PG synthesis remains poorly understood. In this study, we determined the role of DivIVA in regulating lateral PG synthesis of Streptococcus suis and identified a critical interacting partner, MltG, in which DivIVA influenced the subcellular localizations of MltG through its phosphorylation. Our study characterizes the detailed role of DivIVA in regulating bacterial PG synthesis, which is very helpful for understanding the process of PG synthesis in streptococci.

## INTRODUCTION

Bacteria can maintain specific shapes and survive in changing environmental conditions, which is primarily attributed to the presence of peptidoglycan (PG) in the bacterial cell wall. PG has a multilayer structure that is composed of linear glycan strands cross-linked by short peptides, forming a mesh-like sacculus that surrounds the bacterial membrane (1). The sacculus provides a physical barrier that protects bacteria from lysis due to osmotic pressure (2). Considering the important role of PG in bacterial morphogenesis and physiology, as well as its absence from higher eukaryotes, the PG biosynthesis pathway provides a rich source of novel antimicrobial drug targets (3).

The biosynthesis of PG starts from the synthesis of the precursor lipid II in the cytoplasm. The lipid II is then flipped across the cytoplasmic membrane and polymerized and cross-linked to form the PG sacculus (4). In rod-shaped bacteria, such as Escherichia coli, there are two protein complexes elongasome and divisome that direct the synthesis of peripheral PG and septal PG, respectively (5, 6). The elongasome organized by MreB, which is a homolog of eukaryotic actin, is responsible for the PG synthesis along the lateral part of the cell, facilitating the elongation of bacterial cells (7–9). The PG synthesis is shifted to the septum once the cell reaches a certain length, although the mechanism of how this transition is regulated remains unclear. The divisome, featured by FtsZ, a homolog of eukaryotic tubulin, directs cell constriction and PG synthesis in the cell equator leading to cell division (10–12).

Ovoid-shaped bacteria, such as streptococci, also undergo peripheral and septal PG synthesis during cell division and therefore possess elongasome and divisome as well (4, 13). However, MreB is absent from most ovococci, and unlike Bacillus, in which the elongasome and divisome are two distinct complexes, the two protein machinery in ovoid-shaped bacteria are localized closely together at the mid-cell, suggesting that a different mechanism exists to control PG synthesis in ovococci (14). It has been suggested that in Streptococcus pneumoniae, the RodA-PBP2b complex is the core enzyme for peripheral PG synthesis, and the FtsW-PBP2x complex is responsible for the PG synthesis at the septum leading to cell invagination (15). Additionally, several other proteins have been identified as components of elongasome or divisome, although they may have similar subcellular localizations (14). MltG is a recently identified component of elongasome in S. pneumoniae (16). Biochemical data suggest that S. pneumoniae MltG is a PG muramidase rather than a lytic transglycosylase; therefore, it is also named MpgA for membrane-bound peptidoglycan glycosidase.

A key but still unresolved question is how PG synthesis is precisely regulated to generate the ovoid shape. Apart from the enzymes directly involved in PG synthesis, several regulatory proteins have been identified to be closely related to the morphogenesis of ovococci (17). DivIVA is a coiled-coil protein conserved in Gram-positive bacteria but absent from Gram-negative bacteria (18). Previous studies have revealed an important role of DivIVA in the cell division of Bacillus subtilis (19, 20), Listeria monocytogenes (21, 22), Mycobacterium tuberculosis (23), and S. pneumoniae (24, 25). It was originally discovered in B. subtilis, which has the ability to sense membrane curvature (26, 27). By interacting with the Min system through MinJ, DivIVA functions to prevent inappropriate division at the cell poles (28). In S. pneumoniae, which lacks a Min system, DivIVA has also been shown to be critical for cell elongation, as well as cell division (24). Deletion of *divIVA* gene results in significantly rounder cells, suggesting an important role of DivIVA in the regulation of cell elongation in streptococci (24). Peripheral PG synthesis occurs between the future equator and the nascent dividing cell septum (29). Blocking this process by deletion or depletion of cell elongation-related proteins, such as PBP2b and MreCD in S. pneumoniae (17), produces nearly spherical cell chains with a similar appearance. The significantly reduced aspect ratio of *divIVA* deletion mutant (24) suggests that DivIVA might be involved in the synthesis of peripheral PG.

Moreover, eukaryotic serine/threonine kinase (STK)-mediated protein phosphorylation has been reported to have a profound impact on the cell division of streptococci (1). DivIVA has been identified as a prominent substrate of STK (1, 24, 30). In S. pneumoniae strain R800, mutating the phosphorylation site of DivIVA caused abnormal cell morphology in which the cells showed an elongated shape with a polar bulge (31). However, the mechanism of how DivIVA and its phosphorylation regulate cell elongation and daughter cell separation remains to be revealed.

Streptococcus suis is an important zoonotic bacterium, which causes meningitis, arthritis, sepsis, and even death in pigs, leading to huge economic losses in the pig industry worldwide (32). Moreover, it can cause fetal infections in humans, posing a threat to public health (32). Thus, it is of major significance to further study the cell division of S. suis. In this important zoonotic pathogen, we previously revealed the important regulatory roles of STK on growth, cell division, and virulence and identified several STK substrates, including DivIVA (33, 34). In this study, by using transmission electron microscopy (TEM) and fluorescent labeling of nascent PG and bacterial proteins followed by structured illumination microscopy (SIM), we provide detailed evidence that DivIVA mainly functions in the regulation of peripheral PG synthesis, as well as daughter cell separation. We also found that the phosphorylation state of DivIVA is critical for its function. By identifying the interaction partners of DivIVA using coimmunoprecipitation (co-IP) and bacterial two-hybrid, we further reveal the mechanism of how DivIVA exerts its regulatory functions on PG synthesis.

## RESULTS

### DivIVA is critical for peripheral PG synthesis in S. suis.

**FIG 1:**
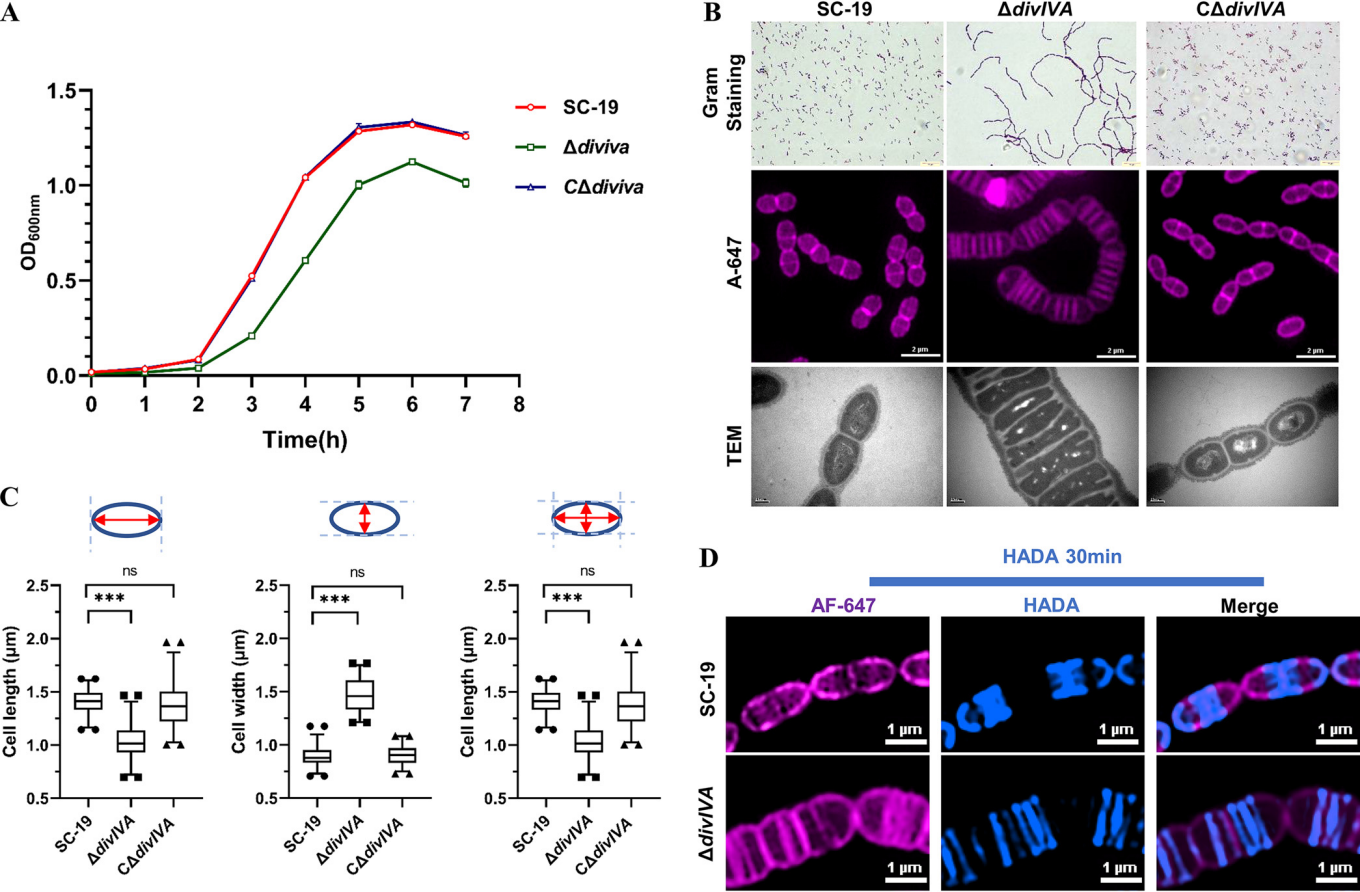
*divIVA* deletion affects growth, morphology, and peripheral PG synthesis of S. suis. (A) Growth curves. Each indicated strain was grown in tryptic soy broth (TSB) medium from the overnight culture at 37°C with shaking. The turbidity (optical density at 600 nm [OD_600_]) of the cultures was measured at 1-h intervals. (B) Morphology examination. (Top) The cells of each indicated strain were grown to the mid-log phase and were subjected to Gram staining. The cells were then imaged using a light microscope. Bar, 20 μm. (Middle) The cells of each indicated strain were grown to the mid-log phase, stained with Alexa Fluor 647 fluorescent dye (A647; violet), and visualized using a structured illumination microscope (SIM). Bar, 2 μm. (Bottom) The cells of each indicated strain were grown to the mid-log phase. Bar, 200 nm. The samples were prepared as described in Materials and Methods and were examined using a transmission electron microscope (TEM). (C) Measurement of cell lengths, widths, and aspect ratios (cell length to width). One hundred or more cells from two independent experiments were measured as described in Materials and Methods for each strain. *P* values were obtained by two-tailed, unpaired Student’s *t* test. ***, *P* < 0.001; ns, no significance. (D) AF-647 membrane staining and HADA labeling of PG. The cells of each indicated strain were grown to the mid-log phase. The cells were then pulsed with HADA for 30 min followed by staining with AF-647 dye. The samples were visualized using a structured illumination microscope (SIM). Bar, 1 μm.

To verify the hypothesis that DivIVA mainly regulates the synthesis of peripheral PG in Streptococcus, we first constructed a *divIVA* deletion mutant (Δ*divIVA*) and its complementary strain (CΔ*divIVA*) in S. suis strain SC-19. Growth assay by measuring the optical density at 600 nm (OD_600_) showed that Δ*divIVA* had a lower growth rate than SC-19 and CΔ*divIVA* (Fig. 1A). Gram-staining analysis showed that Δ*divIVA* cells formed exceptionally long chains compared with the SC-19 and CΔ*divIVA* cells (Fig. 1B). SIM and TEM analyses were performed to further characterize the cell morphology. The chained morphology of Δ*divIVA* was confirmed (Fig. 1B), and it was revealed that Δ*divIVA* cells displayed smaller cell length and larger width, resulting in a significantly decreased aspect ratio, while the complementation strain (CΔ*divIVA*) showed a similar aspect ratio compared with the wild-type (WT) strain (Fig. 1C), indicating that the Δ*divIVA* cells underwent abnormal PG synthesis, as well as defects in daughter cell separation.

To further observe the details of PG synthesis, the fluorescent dye HADA that labels the nascent PG was used to indicate nascent PG synthesis (35). We first used HADA to label the newly synthesized PG of the cells. SIM observation revealed that peripheral PG was synthesized along the longitudinal axis of cells of WT strain SC-19. In contrast, despite the synthesis of normal septal PG, nearly no peripheral PG was synthesized in the Δ*divIVA* cells (Fig. 1D). These data suggest that DivIVA plays an important role in regulating PG synthesis, especially the synthesis of peripheral PG, as well as daughter cell separation.

### DivIVA is phosphorylated by STK on S145, T199, and T211.

**FIG 2:**
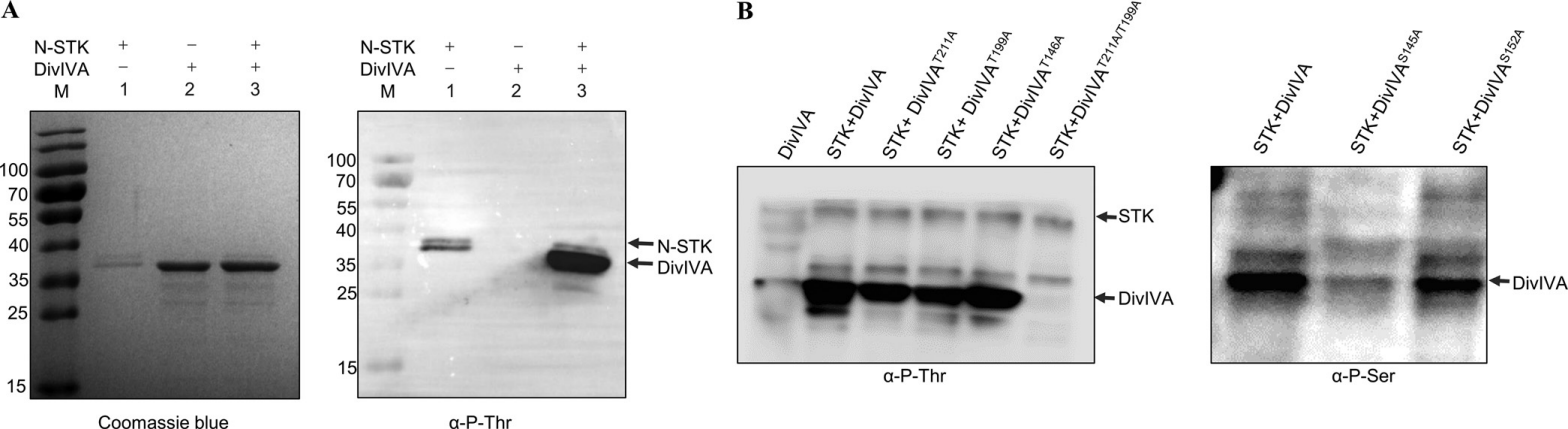
Validation of the phosphorylation sites of DivIVA. (A) *In vitro* phosphorylation of DivIVA by STK. The purified N-terminal domain of STK (N-STK) and DivIVA were incubated together, separated by SDS-PAGE, and stained with Coomassie blue (left) or analyzed by Western blotting with an anti-p-Thr antibody (right). (B) *In vitro* phosphorylation of the DivIVA variant by STK. DivIVA and its variant with the point mutations were purified and incubated with STK. The samples were resolved by SDS-PAGE followed by Western blotting with anti-pThr and anti-pSer antibodies, respectively.

In our previous study for the function of S. suis STK, DivIVA was identified as a substrate of STK, and two potential phosphorylation sites (T199 and T211) were revealed by comparative phosphoproteomic analysis (34). To further confirm the phosphorylation sites in DivIVA, an *in vitro* phosphorylation assay was conducted. It was shown in Fig. 2A that a strong signal was observed when purified STK and DivIVA proteins were incubated together and probed with the anti-P-Thr antibody, suggesting that DivIVA can be phosphorylated by STK. The phosphorylation sites of DivIVA were further analyzed by high-performance liquid chromatography mass spectrometry (HPLC-MS), and the results showed that T199, T211, and another residue among S145, T146, and S152 were the potential phosphorylation sites (Table 1; Fig. S1A). We next purified the DivIVA variants containing the substitutions T199A, T211A, S145A, T146A, S152A, and T199A-T211A, respectively, and performed the *in vitro* phosphorylation assay with STK again. The Western-blot results using the anti-P-Ser or anti-P-Thr antibody showed that the phosphorylation of DivIVA by STK was almost abolished in the case of T199A to T211A substitutions and the S145A substitution. These results suggest that DivIVA is a substrate of STK, and the phosphorylation sites are the residues S145, T199, and T211.

**TABLE 1:** Identification of phosphorylation sites of DivIVA^*a*^

Phosphorylated tryptic peptide sequence of DivIVA phosphorylated by purified STK from pET-28a^*b*^	No. of phosphate groups detected by LC-MS/MS	Phosphorylated residue
[184–212]ALDEELPVEEESLDY**pT**RQLTPEEIAELTR	1	T199
[201–212]QLTPEEIAEL**pT**R	1	T211
[145–178]**pSpT**VESQL**pS**LVNSSEWEEILRPTASYIQTSDEAFR	1	S145, T146, or S152

a LC-MS/MS, liquid chromatography-tandem mass spectrometry; STK, serine/threonine kinase.

b Sequences of the phosphorylated peptides identified in DivIVA as determined by LC-MS/MS following tryptic digestion are indicated, and phosphorylated residues (pT or pS) are shown in bold, underlined type. In peptide [145–178], only one phosphorylation site was detected, which was one of S145, T146, and S152.

### DivIVA phosphorylation is essential for morphology maintenance and PG biosynthesis of S. suis.

**FIG 3:**
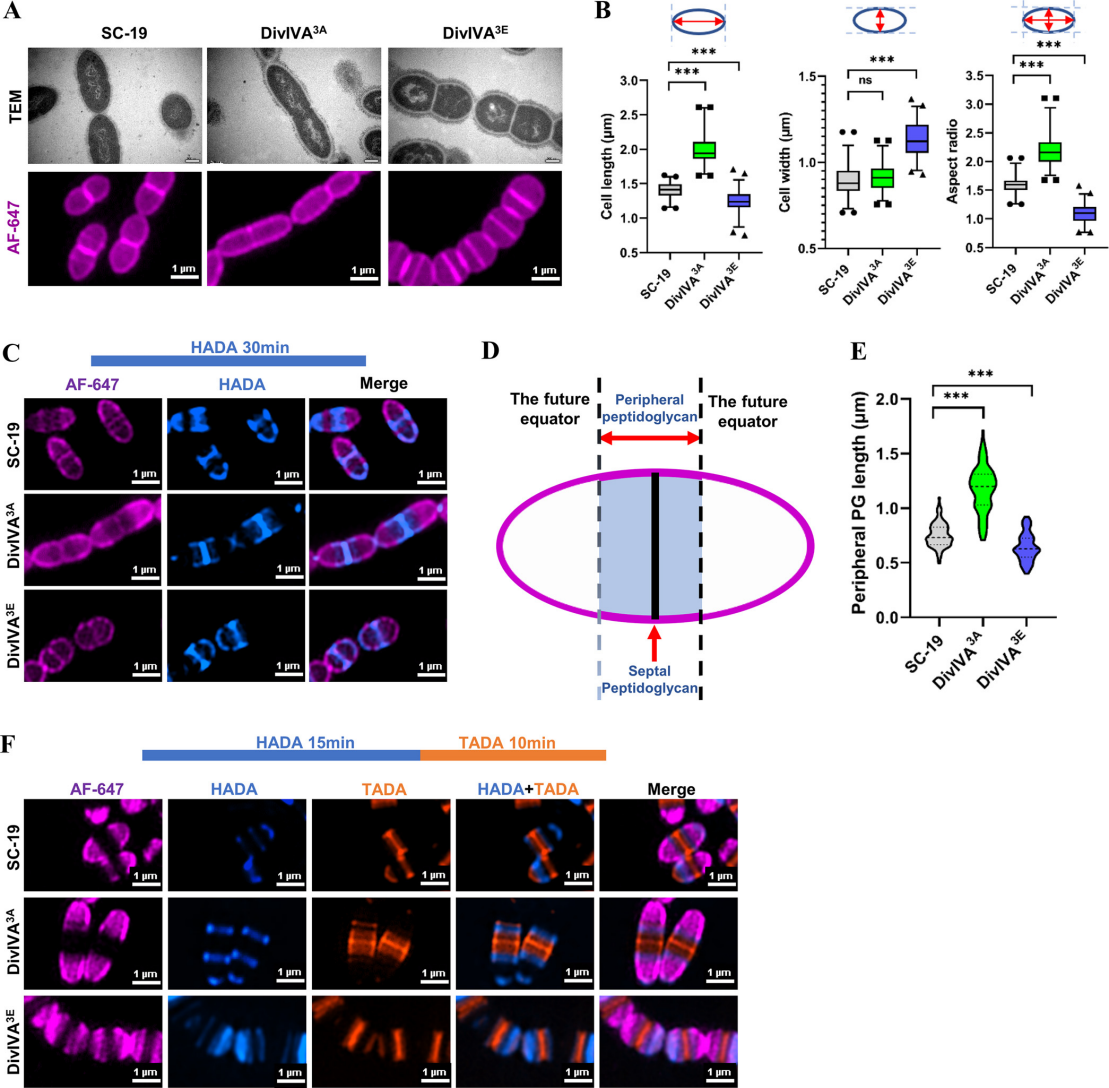
Influence of DivIVA phosphorylation on morphology and PG synthesis of S. suis. (A) Influence of DivIVA phosphorylation on morphology. Cells of S. suis strain SC-19, DivIVA^3A^ (DivIVA^S145A/T199A/T211A^), and DivIVA^3E^ (DivIVA^S145E/T199E/T211E^) were grown to the mid-log phase and subjected to analysis by TEM (upper) or by SIM following AF-647 staining (lower). Bar, 200 nm for TEM images and 1 μm for SIM analysis. (B) Measurement of cell lengths, widths, and aspect ratios (cell length to width ratio). One hundred or more cells from two independent experiments were measured as described in Materials and Methods for each strain. The *P* values were obtained by two-tailed, unpaired Student’s *t* test. ***, *P* < 0.001; ns, no significance. (C) Visualization of nascent peptidoglycan (PG). Cells of S. suis strain SC-19, DivIVA^3A,^ and DivIVA^3E^ were grown to the mid-log phase and incubated with HADA for 30 min followed by staining with AF-647 dye and were then subjected to SIM analysis. Bar, 1 μm. (D) A model showing the peripheral PG. (E) Violin plot of nascent PG length. Cells of S. suis strain SC-19, DivIVA^3A,^ and DivIVA^3E^ were grown to the mid-log phase and incubated with HADA for 30 min followed by staining with AF-647 dye. One hundred or more cells were selected for measurement as described in Materials and Methods. *P* values were obtained by two-tailed, unpaired Student’s *t* test. ***, *P* < 0.001. (F) Pulse-chase assay with two different dyes. HADA was first used to stain the log-phase cells for 10 min and were then chased with TADA for 30 min. The PG-labeled cells were washed and then stained with AF647. The cells were imaged using a structured illumination (SIM) microscope.

In order to assess the role of DivIVA phosphorylation in the cell division of S. suis, two mutant S. suis strains were constructed in which the native *divIVA* gene in the chromosome was replaced with DivIVA^3A^ (DivIVA^S145A-T199A-T211A^) containing the three substitutions with alanine (A) mimicking the dephosphorylated form, and DivIVA^3E^ (DivIVA^S145E/T199E/T211E^) containing the three substitutions with glutamate (E) mimicking the phosphorylated form, respectively. The morphology of the two mutant strains was analyzed with SIM following AF647 staining. It was shown that cells of the DivIVA^3A^ strain were significantly longer than those of SC-19 or the DivIVA^3E^ strain (Fig. 3A and B). In contrast, the DivIVA^3E^ strain displayed shorter but wider cells than SC-19 (Fig. 3A and B). These data demonstrate that DivVIA phosphorylation plays an important role in controlling the cell morphology of S. suis.

To further dissect the influence of DivVIA phosphorylation on the morphogenesis of S. suis, we used d-amino acid fluorescent probe HADA to label the nascent PG to observe the effect of DivIVA phosphorylation on PG synthesis. First, cells of SC-19, DivIVA^3A^, and DivIVA^3E^ were stained with HADA for 5 min followed by SIM analysis. It was shown that the nascent PG was synthesized at the mid-cell in all three strains, indicating that the phosphorylation state of DivIVA did not affect the site of nascent PG synthesis (Fig. S2). Then, HADA incubation time was extended to 30 min (approximately three-quarters to one cell cycle), and the cells were subjected to PG length measurement. Normally, in S. pneumoniae, peripheral PG is first synthesized to elongate the cell, followed by simultaneous synthesis of peripheral and septal PG. Then, PG was cleaved at the septum, leading to cell invagination and daughter cell separation at the mid-cell (36). Therefore, the cells that had not started invagination were selected for peripheral PG length measurement. It was shown that the peripheral PG labeled in DivIVA^3A^ strain was significantly longer than that of the SC-19 strain, while the nascent peripheral PG in DivIVA^3E^ was significantly shorter (Fig. 3C and E). To further characterize the PG synthesis, a pulse-chase experiment in which the HADA dye (blue) was first used to stain the cells for 10 min followed by a chase with TADA dye (orange) for 30 min. By SIM analysis, it was shown that DivIVA^3A^ cells displayed significantly longer peripheral PG, which was shorter in DivIVA^3E^ cells compared with the WT cells (Fig. 3F). The results suggest that DivIVA phosphorylation does regulate the peripheral PG synthesis.

### DivIVA interacts with the cell wall hydrolase MltG.

**FIG 4:**
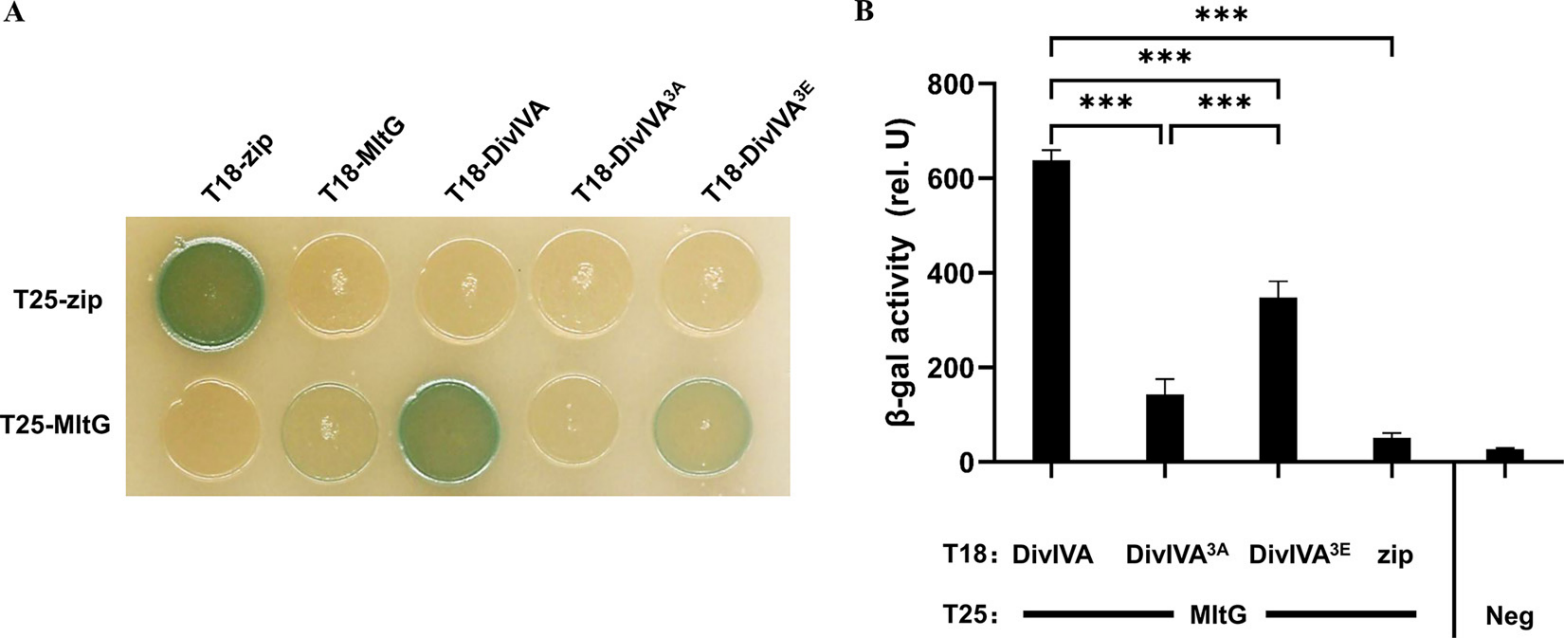
Interaction between MltG and DivIVA determined by bacterial two-hybrid. (A) Bacterial two-hybrid assay. E. coli BTH101 (*Δcya*) carrying pUT18 or pKNT25 expressing the indicated protein (T25 and T18) were grown to the mid-log phase, and 5 μL was spotted on LB agar plates containing X-gal, incubated at 30°C, and imaged. pT18-zip and pT25-zip were used as the positive control. (B) Quantitative analysis of the protein interaction. The cells harboring the pUT18- and pKNT25-derived plasmids were incubated at 30°C for 16 h. The cells subjected to β-galactosidase activity measurement as described in Materials and Methods.

To address how DivIVA regulates PG synthesis of S. suis, a co-IP/MS assay was conducted to identify the interacting partners of DivIVA protein. The cell lysate of strains SC-19 and Δ*divIVA* was incubated with the anti-DivIVA antibody, respectively, followed by precipitation with protein A beads. The coimmunoprecipitated proteins and the proteins in the control that nonspecifically binds to the beads were subjected to MS analysis to compare the protein abundance. As shown in Table 2, a total of 41 proteins showed a significant difference in enrichment and were regarded as the potential proteins interacting with DivIVA. These proteins include MltG, a recently identified member of elongasome involved in cell extension, and its deletion makes cells shorter and suppresses the need for PBP2b protein (37). We next verified the DivIVA-MltG interaction, using a T18-T25-based bacterial two-hybrid (BTH) system. If there is an interaction between the two proteins, T18 and T25 recombine the cAMP-producing adenylate cyclase, thereby inducing the expression of β-galactosidase, which cleaves 5-bromo-4-chloro-3-indolyl-β-d-galactopyranoside (X-gal) in the medium, appearing in blue. The results showed that DivIVA could interact with MltG (Fig. 4A). Moreover, it was shown that the dephosphorylated form DivIVA^3A^ did not interact or had a very weak interaction with MltG (but could interact with other unrelated proteins, excluding the possibility of protein instability or expression problem [Fig. S3]), while the phosphomimetic form DivIVA^3E^ still did (Fig. 4A). Quantitative analysis by β-galactosidase activity measurement further revealed that DivIVA^3A^ had a significantly weaker interaction with MltG than DivIVA^3E^ (Fig. 4B). These data suggest that DivIVA phosphorylation may regulate peripheral PG synthesis through MltG.

**TABLE 2:** Putative DivIVA-interacting proteins^*a*^

Accession number	Locus	Gene	Protein function	Predicted molecular mass (kDa)	SC-19/Δ*divIVA*	
					Log_2_ (ratio)	Sig
A4VTL4	SSU05_0487	*divIVA*	Cell division initiation protein	26.212	14.1447	++
A4VW88	SSU05_1411	*ftsE*	Cell division ATP-binding protein	27.698	2.7083	++
A4VXD5	SSU05_1808		Transcriptional regulator	16.691	2.6284	++
A4VSS2	SSU05_0193		phosphoric diester hydrolase activity	67.728	2.2408	++
A4VX44	SSU05_1717	*mltG*	Endolytic murein transglycosylase	40.277	2.2039	++
A4VTU3	SSU05_0566	*cpsD*	Tyrosine-protein kinase	25.036	2.1868	++
A4VY72	SSU05_2095		Putative cyclo-nucleotide phosphodiesterase	88.162	2.018	++
A4VVU4	SSU05_1267		Type IIA topoisomerase	95.484	2.0116	++
A4VUK3	SSU05_0826		Uncharacterized protein conserved in bacteria	14.605	1.9804	++
A4VXU5	SSU05_1968		DNA nuclease	113.94	1.9637	++
A4VVU6	SSU05_1269	*rpmI*	50S ribosomal protein L35	7.6911	1.9	++
A4VW87	SSU05_1410	*ftsX*	Cell division protein FtsX	35.393	1.8979	++
A4VSW9	SSU05_0240	*msrA*	Multifunctional fusion protein	35.624	1.7385	+
A4VSW2	SSU05_0233		Uncharacterized protein	11.958	1.7161	+
A4VX45	SSU05_1718		Predicted periplasmic solute-binding protein	25.175	1.6981	+
A4VTI3	SSU05_0456		Superfamily II helicase	13.908	1.6915	+
A4VT05	SSU05_0276	*rpmG*	50S ribosomal protein L33	5.9249	1.6104	+
A4VU28	SSU05_0651	*murE*	UDP-*N*-acetylmuramoyl-l-alanyl-d-glutamate-l-lysine ligase	53.209	1.6102	+
A4VX26	SSU05_1699		Pseudouridine synthase	27.371	1.5572	+
A4VSE1	SSU05_0058		Heme/copper-type cytochrome/quinol oxidase, subunit 1	58.375	1.5452	+
A4VVT0	SSU05_1253		ABC-type uncharacterized transport system, ATPase component	29.384	1.5222	+
A4VWI5	SSU05_1508		Phosphoserine phosphatase	24.531	1.4227	+
A4VUE9	SSU05_0772		Putative ribosomal protein S1-like DNA-binding protein	4.8867	1.4075	+
A4VSH2	SSU05_0089	*rplO*	50S ribosomal protein L15	17.516	1.4002	+
A4VWQ4	SSU05_1577		Putative internalin A	90.926	1.3752	+
A4VWK7	SSU05_1530		Integrase	5.3521	1.3538	+
A4VTU2	SSU05_0565	*cpsC*	Capsular polysaccharide biosynthesis protein	25.152	1.3465	+
A4VVU8	SSU05_1271	*cmK*	Cytidylate kinase	24.717	1.3436	+
A4VW48	SSU05_1371		Ribonucleases G and E	67.175	1.3352	+
A4VX94	SSU05_1767		Uncharacterized conserved protein	26.288	1.3227	+
A4VW38	SSU05_1361		ABC-type polar amino acid transport system, ATPase component	28.118	1.3125	+
A4VVY0	SSU05_1303		GTP cyclohydrolase 1 type 2 homolog	29.39	1.2853	+
A4VTS2	SSU05_0545		Methyl-accepting chemotaxis protein	123.71	1.2546	+
A4VTN7	SSU05_0510		Uncharacterized protein	28.076	1.2041	+
A4VTJ4	SSU05_0467		Aspartate-ammonia ligase	35.73	1.1681	+
A4VYF2	SSU05_2175	*ecfA2*	ATP-binding protein	30.801	1.1375	+
A4VV27	SSU05_1000		Putative 5-nucleotidase	60.776	1.119	+
A4VWM2	SSU05_1545		ABC-type branched-chain amino acid transport system	4.4281	1.1025	+
A4VTB0	SSU05_0382		Uncharacterized protein	91.444	1.0691	+
A4VXJ3	SSU05_1866		ABC-type oligopeptide transport system, ATPase component	35.668	1.0674	+
A4VUH7	SSU05_0800		ABC-type multidrug transport system, ATPase and permease component	35.682	1.0639	+
A4VWL5	SSU05_1538		Putative 5-nucleotidase	72.8	1.0356	+

a The coimmunoprecipitation (co-IP) was carried out as described in the Materials and Methods. The ratio in the sixth column indicates the ratio of the number of peptides immunoprecipitated from the S. suis SC-19 cells to those from Δ*divIVA* (control). The normal distribution was fitted, and the means ± 1.64*SD were calculated, with 1 and mean + 1.64*SD taken as the threshold of Log_2_(ratio). The classification is as follows: ++, Log_2_(ratio) ≥ (mean + 1.64*SD); +, (mean + 1.64*SD) > log_2_(ratio) ≥ 1.

### DivIVA does not affect the PG hydrolase activity of MltG.

**FIG 5:**
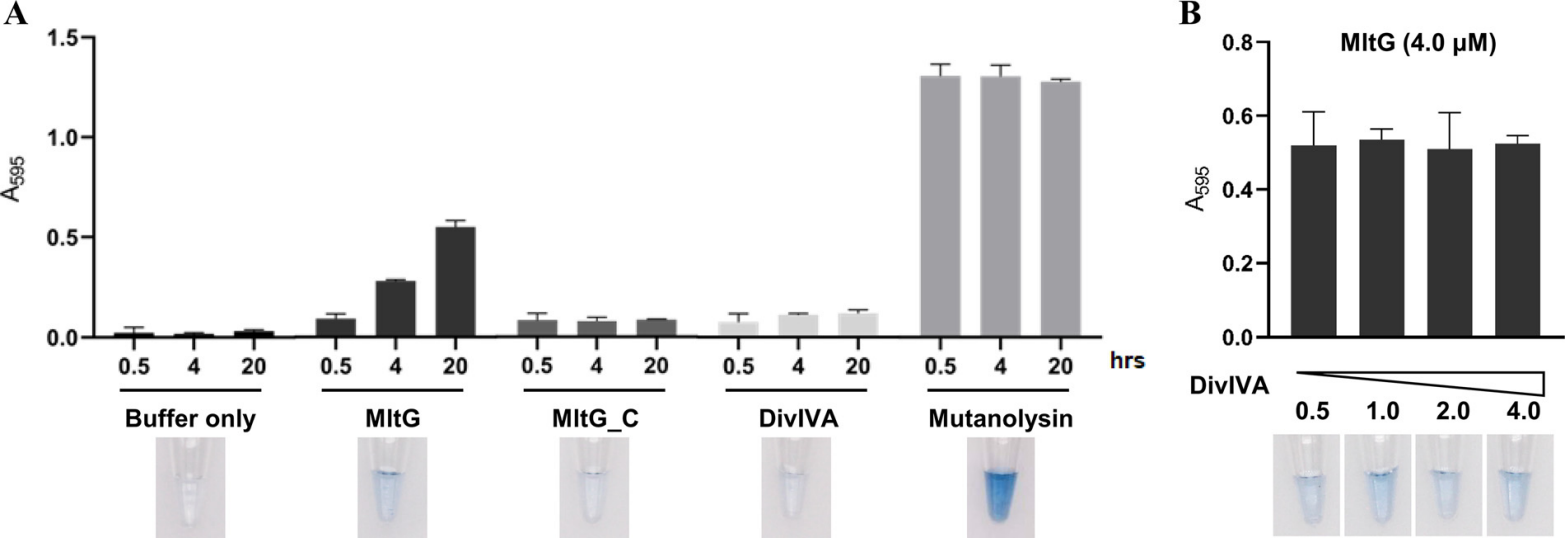
Influence of DivIVA on the activity of MltG. (A) Determination of the PG hydrolase activity of MltG. Remazol brilliant blue (RBB)-labeled peptidoglycan was incubated with MltG (4 μM), MltG_C (4 μM), DivIVA (4 μM), mutanolysin (10 U), or buffer, respectively, at 37°C. At each time point, the reaction was terminated, and the supernatant of the sample was subjected to measurement of absorbance at 595 nm. The pictures provided below the panel showed the results with an incubation of 20 h. Three replicate experiments were performed for each reaction, with the means ± standard deviations presented. (B) Influence of DivIVA on the activity of MltG. RBB-labeled peptidoglycan was incubated with MltG, as well as different concentrations of DivIVA. The absorbance at 595 nm was measured. Three replicate experiments were performed for each reaction, with the means ± standard deviations presented.

MltG was recently shown as a PG muramidase in S. pneumoniae (38), and the enzymatic activity was critical for its function (16). We then constructed S. suis strain MltG^1-500^, in which the catalytic domain was truncated, and strain MltG^N507D^, in which the enzymatic activity site (N507) was mutated. By SIM analysis following by AF-647 staining, it was shown that similar to Δ*divIVA* cells, the cells of MltG^1-500^ and MltG^N507D^ showed smaller length and bigger width, displaying a significantly rounder morphology (Fig. S4), indicating that the catalytic activity of MltG is involved in the morphology regulation of S. suis. Next, we examined whether DivIVA has any impact on the catalytic activity of MltG. We used the dye Remazol brilliant blue (RBB) to label the hydrolyzed peptidoglycan of S. suis, which can indicate the PG hydrolase activity by measuring the absorbance (39, 40). As shown in Fig. 5A, the absorbance value of the stained peptidoglycan solution treated with mutanolysin (positive control) increased significantly within 0.5 h and then was maintained at a high level at 4 and 20 h postreaction without significant change. We then tested the PG hydrolase activity of MltG, the carboxyl-terminal domain of MltG (MltG_C), and DivIVA. It was shown that MltG, but not the other two proteins, exhibited PG hydrolase activity (Fig. 5A), suggesting that MltG of S. suis is a PG hydrolase. We next measured the PG hydrolase activity of MltG in the presence and absence of DivIVA to see whether DivIVA interferes with the activity of MltG. As shown in Fig. 5B, the absorbance values did not show significant change when different amounts of DivIVA were added, suggesting that the interaction between DivIVA and MltG does not affect the peptidoglycan hydrolase activity MltG.

### DivIVA phosphorylation affects MltG localization and inhibits peripheral PG synthesis.

**FIG 6:**
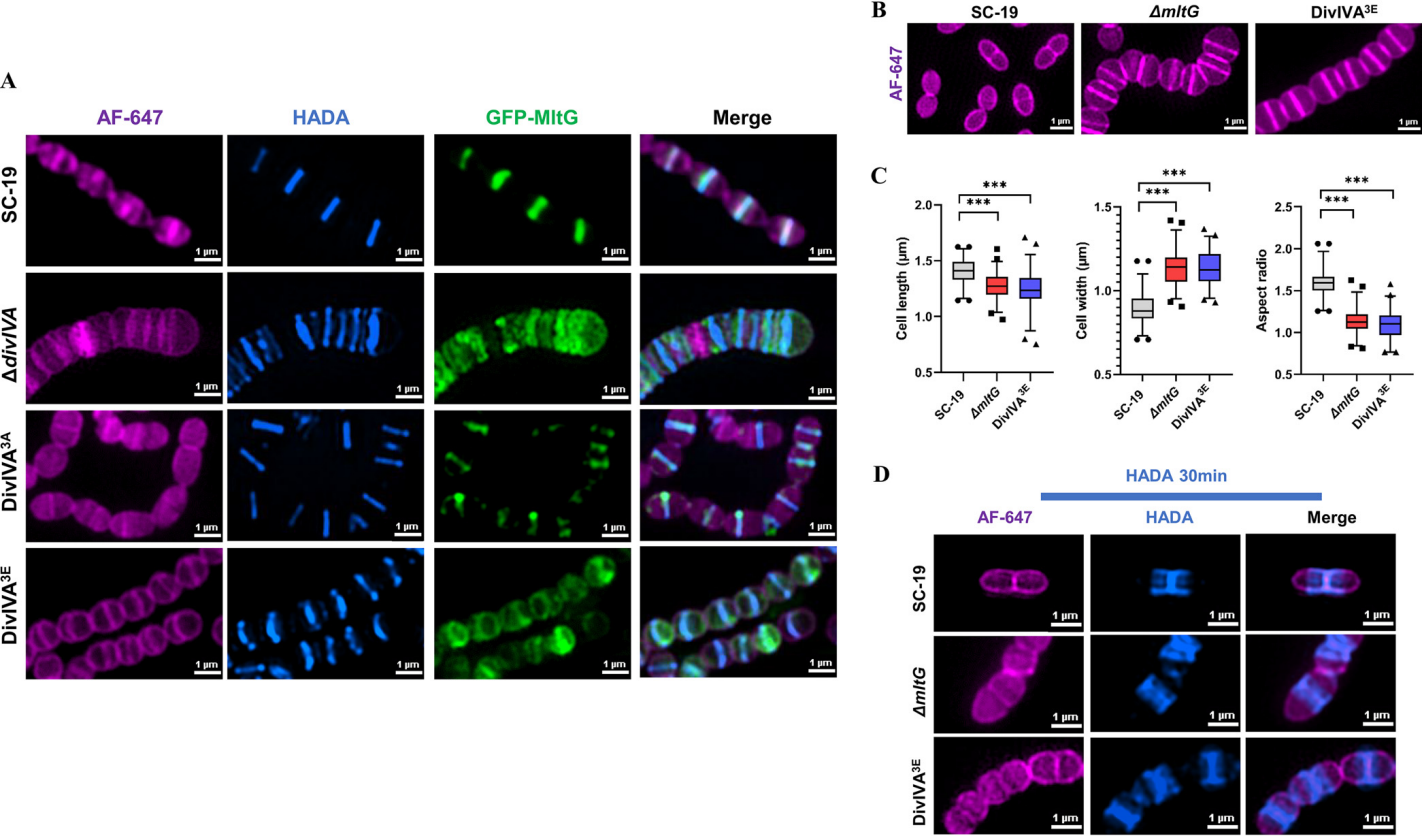
DivIVA phosphorylation affects MltG localization and peripheral PG synthesis. (A) Localization of MltG in th*e* S. suis SC-19, Δ*divIVA*, DivIVA^3A^, and DivIVA^3E^ strains. The cells of each indicated strain were grown in the presence of 0.3 mM tetracycline to induce GFP-MltG expression to the log-phase cells, which were then pulsed with HADA for 5 min at 37°C in TSB medium. The cells were imaged using a SIM. Bar, 1 μm. (B) Imaging of SC-19, Δ*mltG*, and DivIVA^3E^ following AF-647 staining. The cells of each indicated strain were grown to the mid-log phase and stained with AF-647 dye at 37°C in TSB medium. The samples were visualized by 3D-SIM. Bar, 1 μm. (C) Box-and-whisker plots of cell lengths, widths, and aspect ratios (cell length to width) of SC-19, Δ*mltG*, and DivIVA^3E^. One hundred or more cells from two independent experiments were measured as described in Materials and Methods. The *P* values were obtained by two-tailed, unpaired Student’s *t* test. ***, *P* < 0.001. (D) Visualization of nascent PG. Cells of SC-19, Δ*mltG*, and DivIVA^3E^ were grown to the mid-log phase, incubated with HADA for 30 min followed by staining with AF-647 dye, and then subjected to SIM analysis. Bar, 1 μm.

To examine the influence of DivIVA on subcellular localization of MltG, a green fluorescent protein (GFP)-tagged MltG (GFP-MltG) was expressed in SC-19, Δ*divIVA*, DivIVA^3A^, and DivIVA^3E^ strains, respectively, and the MltG localization was determined by AF647 labeling followed by SIM imaging. As shown in Fig. 6A, GFP-MltG was seen at the mid-cell of the SC-19 cells, consistent with its localization in S. pneumoniae reported previously (16). However, GFP-MltG was more dispersedly distributed in the Δ*divIVA* cells. In addition, in the DivIVA^3E^ strain where the DivIVA phosphorylation sites were substituted with glutamate mimicking a phosphorylated state, the mid-cell localization of GFP-MltG was also impaired in contrast to DivIVA^3A^ in which the GFP-MltG localization was normal, suggesting that DivIVA and its phosphorylation are critical for proper localization of MltG. We further constructed an *mltG* deletion mutant of S. suis (Δ*mltG*). Morphological analysis under SIM following AF647 staining revealed that compared with the SC-19 cells that formed a typical ovoid shape, both Δ*mltG* and DivIVA^3E^ cells were significantly shorter and wider (Fig. 6B and C). Imaging of nascent PG synthesis by HADA staining further revealed that Δ*mltG* and DivIVA^3E^ cells undertook impaired peripheral PG synthesis (Fig. 6D). This indicates that *divIVA* deletion leads to abnormal localization of the interacting protein MltG, which in turn leads to the dysfunction of the elongasome, resulting in the shortening of Δ*divIVA* cells. When DivIVA is phosphorylated, it interacts with MltG, causing abnormal localization of MltG and terminating the synthesis of peripheral PG. Together, we demonstrate that DivIVA phosphorylation maintains normal cell morphology by affecting MltG localization, terminating the peripheral PG synthesis in the initial stage.

## DISCUSSION

The precise regulation of PG synthesis to produce the ovoid shape is a crucial yet unanswered question. In additino to the enzymes involved in PG synthesis, a number of regulatory proteins have been found to be intimately linked to ovococci morphogenesis (14, 17). DivIVA is a coiled-coil tropomyosin-like protein present only in Gram-positive bacteria (41), and its amino-terminal domain (connected to the cell membrane) is strictly conserved, while the carboxyl-terminal domain is not conserved (42). As a result, the DivIVA protein’s function varies according to the species of bacteria. In bacteria such as B. subtilis, it forms the Min system together with MinCD, which regulates FtsZ (Z-ring) assembly and localization (28). Deletion of DivIVA results in defective cell separation in the rod-shaped bacterium L. monocytogenes. The cell segregation defect was attributed to autolysin p60 and MurA blocked by *divIVA* deletion through the SecA2 secretion pathway (22). In mycobacteria, DivIVA, also known as Wag31, is essential and localizes to the septum, cytopoles, and branching sites and plays a role in promoting PG synthesis at the cell poles (23). In this study, we found that DivIVA was essential for the peripheral PG synthesis in S. suis. The synthesis process of nascent PG in the *divIVA* deletion cells was observed by HADA probing and super-resolution 3D-SIM microscopy. Deletion of *divIVA* caused a nearly complete halt in peripheral PG synthesis, resulting in noticeably shorter and flatter cells. This implies that DivIVA is one of the components of S. suis elongasome. However, in S. pneumoniae, the absence of DivIVA also results in cells showing compressed round cells (24), so we speculate that DivIVA also regulates the synthesis of peripheral PG in some ovoid-shaped bacteria.

Although several proteins are known to be involved in the regulation of PG synthesis (24, 43–46), little is known about how the elongasome and divisome collaborate temporally and spatially to maintain normal cell shape and size. In recent reports, eukaryotic-type serine/threonine kinase STK plays a key role in coordinating the synthesis of peripheral PG and septal PG (1, 31, 47). Several morphogenetic proteins, including DivIVA, EloR, FtsZ, MacP, and MapZ, were found to be the substrates of STK (24, 48–51). In addition, disrupting the normal phosphorylation of some STK substrates leads to PG biosynthesis disorder and morphological defects (31, 43). Importantly, in S. suis, STK phosphorylates DivIVA at S145, T199, and T211, whereas in S. pneumoniae, DivIVA has only one phosphorylation site, T201, which is phosphorylated by STK (50). Through morphological studies, our study found that the phosphorylation-depleted mutant DivIVA^3A^ (DivIVA^S145A/T199A/T211A^) cells were significantly longer than the wild-type cells, whereas the phosphorylation-mimicking mutant DivIVA^3E^ (DivIVA^S145E/T199E/T211E^) cells were significantly shorter than the wild type. Phosphorylation of DivIVA had a significant effect on cell length, and this clear morphological change was due to differences in peripheral PG synthesis (Fig. 3D). It is indicated that DivIVA phosphorylation affects cell morphology by regulating the synthesis of peripheral PG, and we infer that DivIVA may terminate the peripheral PG synthesis after being phosphorylated by STK. Since the synthesis of peripheral PG still exists in the DivIVA^3E^ cells (Fig. 3D), we speculate that DivIVA phosphorylation terminates the synthesis of sole peripheral PG before septal PG synthesis is initiated. For S. pneumoniae, R800, a variant strain DivIVA^T201A^ in which DivIVA cannot be phosphorylated, displays an elongated cellular phenotype, with 31% of cells forming a giant polar bulge (31), whereas the variant strains DivIVA^T201A^ and DivIVA^T201E^ display wild-type morphologies in S. pneumoniae R6 (17). The function of DivIVA phosphorylation in S. pneumoniae has not been well elucidated due to the different genetic backgrounds of the strains. In S. suis, DivIVA protein phosphorylation clearly regulates the synthesis of peripheral PG and maintains the ovoid shape. The difference in phosphorylation sites on the DivIVA of S. suis and S. pneumoniae may be the reason for the different phosphorylation functions of DivIVA.

In S. pneumoniae, MltG is a recently reported cell wall hydrolase whose depletion shortens cells while suppressing the requirement for PBP2b, MreCD, RodA, and RodZ, which colocalize with MreC in the septum (16). These together indicate that MltG is involved in cell elongation. Although the precise role of MltG in cell division is uncertain, it has been postulated that it releases PG strands made by PBP1a for cross-linking by RodA/PBP2b (16). In S. suis, through co-IP and bacterial two-hybrid experiments, we found that DivIVA could interact with MltG. Therefore, DivIVA regulates the synthesis of peripheral peptidoglycan which may be related to its interaction with protein MltG. Although MltG is reported as an endolytic transglycosylase in E. coli, it has been recently reported as a PG muramidase in S. pneumoniae (38). By using a RBB-based assay to evaluate its activity for PG cleavage, we showed that MltG of S. suis harbors the PG hydrolase activity. Therefore, we first tested whether DivIVA has an impact on the enzymatic activity of MltG through their interaction. The results showed that the presence of DivIVA does not affect PG hydrolysis of MltG (Fig. 6), indicating interaction DivIVA regulates the peptidoglycan synthesis through other mechanisms. Interestingly, we found that the phosphorylation state of DivIVA affects its interaction with MltG: the dephosphorylated form DivIVA^3A^ could not interact with MltG, while the phosphomimetic form DivIVA^3E^ still could. Moreover, the MltG protein localized at the center of the wild-type cell septum was significantly mislocalized in the Δ*divIVA* and DivIVA^3E^ strains, while it was normally localized in the DivIVA^3A^ strain. This suggested that DivIVA phosphorylation changes the interaction with MltG and thus changes the MltG localization. We hypothesize that after the DivIVA protein is phosphorylated by STK *in vivo*, it interacts with MltG, mislocalizing MltG, terminating peripheral PG synthesis before septal PG synthesis begins, and instead synthesizing septum PG.

Represented by S. pneumoniae, in the early stage of ellipsoid cell division, the cell synthesizes peripheral PG alone for a short time to extend the cell before septal PG synthesis is initiated (29, 52), and then septal and peripheral PG syntheses occur simultaneously in the center of the cell, causing cell invagination. However, this transition from the alone synthesis of peripheral PG to the simultaneous synthesis of septal and peripheral PG may be tightly controlled to maintain proper cell size and shape. In S. suis and S. pneumoniae, DivIVA and EloR are the substrates of STK (34, 50). Cells expressing the dephosphorylated form of EloR (EloR^T89A^) in S. pneumoniae are significantly shorter than wild-type cells, while the phosphor-mimetic form of EloR (EloR^T89E^) was found to overstimulate elongation (43). In contrast, in S. suis, the dephosphorylated form DivIVA^3A^ cells were significantly longer than wild-type cells, while the mimic phosphorylated form DivIVA^3E^ cells were significantly longer than wild-type cells. Interestingly, both EloR and DivIVA can interact with MltG, and DivIVA phosphorylation can alter its interaction with MltG. Therefore, we speculated that STK first phosphorylates EloR, activates the enzymatic activity of MltG, and initiates the synthesis of peripheral PG before septal PG synthesis is initiated. After the peripheral PG is synthesized alone to extend the cell to a certain length, STK phosphorylates DivIVA, and the phosphorylated DivIVA interacts with MltG to change its localization, terminating the synthesis of preseptal peripheral PG. The septal and peripheral PG are then synthesized in the center of the cell simultaneously to invaginate the cell. Although the cells of DivIVA^3E^ and Δ*mltG* strains were shortened, peripheral PG could be synthesized simultaneously with septal PG (Fig. 6C), so the reason for the morphological change was more inclined to the synthesis of preseptal peripheral PG. MltG, a PG muramidase that acts directly on PG (40), is likely to be a target of the STK phosphorylation substrates DivIVA and EloR to regulate preseptal peripheral PG synthesis.

MltG is a potential substrate of the kinase STK in both S. pneumoniae and S. suis according to phosphoproteomic data (34, 53). The biological function of MltG phosphorylated by STK may be related to the regulation of MltG enzyme activity. Therefore, the role of MltG in the synthesis of PG in ovococci needs further study.

In the present study, a direct link between the STK regulatory network and PG synthesis was revealed, and phosphorylation of STK and its substrates plays a key role in the switching and balance of septal and peripheral PG synthesis. However, the time sequence of STK phosphorylation of its substrates and the role of each substrate in PG synthesis remain to be further confirmed.

## MATERIALS AND METHODS

### Bacterial strains, plasmids, and growth conditions.

The bacterial strains and plasmids used in this paper are listed in Table S1. S. suis SC-19 (the wild-type strain, serotype 2) was isolated from a sick pig during a zoonotic epidemic outbreak of S. suis infections in Sichuan Province, China; its genome sequence GenBank accession number is NZ_CP020863.1 (54). S. suis and its derivatives were grown at 37°C in tryptic soy broth (TSB) (Becton, Dickinson, Sparks, NV, USA) supplemented with 7.5% inactivated newborn bovine serum (Sijiqing, Hangzhou, China). E. coli strains were grown in Luria-Bertani (LB) broth at 37°C with shaking. If necessary, chloramphenicol (25 μg/mL), kanamycin (25 μg/mL), spectinomycin (100 μg/mL), streptomycin (20 μg/mL), or erythromycin (90 μg/mL) was supplemented.

### Construction of plasmids, mutants, and complemented strains.

All of the primers used in this paper are listed in Table S2. Unless otherwise specified, the construction of all plasmids was performed using the ClonExpress MultiS one-step cloning kit (Vazyme, Nanjing, China) to ligate fragments into digested plasmids by seamless cloning. The *divIVA* and *mltG* genes of S. suis SC-19 were deleted by a double-crossover approach by homologous recombination (55), resulting in deletion mutants of *divIVA* (Δ*divIVA*) and *mltG* (Δ*mltG*). In order to construct the complementary strain CΔ*divIVA* and CΔ*mltG*, plasmid P*_divIVA_*-*divIVA* or P*_mltG_*-*mltG* was transformed into Δ*divIVA* and Δ*mltG* cells, respectively (56). For the construction of the point mutant strains DivIVA^3A^ (DivIVA^S145A-T199A-T211A^) and DivIVA^3E^ (DivIVA^S145E/T199E/T211E^), the point mutant gene fragment of *divIVA* was first constructed by overlap extension PCR, and then the gene fragment was ligated with the upstream and downstream fragments of the *divIVA* gene and introduced into Δ*divIVA* strain to obtain the point mutant strains DivIVA^3A^ and DivIVA^3E^ by homologous recombination. To construct the MltG^1-500^ strain in which the catalytic domain of MltG was truncated and the MltG^N507D^ strain in which the enzymatic activity site of MltG was mutated, the DNA fragment containing the *mltG* promoter followed by the coding sequence for the MltG variant was cloned into the pSET2 vector, and the recombinant plasmids were then introduced into the Δ*mltG* cells, respectively.

### Protein expression and purification.

pET-28a was used as the vector for His-tagged protein expression with E. coli BL21(DE3) as the host strain. Expression of the protein was induced with 1 mM isopropyl-d-thiogalactopyranoside (IPTG) at 28°C for 8 h. According to the manufacturer’s instructions, His-tagged proteins were purified using nickel-nitrilotriacetic acid (Ni-NTA) columns (GE Healthcare). Ultra centrifugal filters (Millipore) were used to desalt the purified protein in phosphate-buffered saline (PBS) buffer. The quality and amount of isolated proteins were evaluated by using SDS-PAGE and a Micro BCA protein assay kit (Cwbiotech), respectively. The purified protein was then kept at 80°C for storage.

### Protein kinase assay.

*In vitro* kinase assay was performed as previously described (33, 57). The kinase assays were carried out in 50 μL of kinase buffer (50 mM HEPES, pH 7, 1 mM DTT, and 0.01% Brij35) containing 2 mM MnCl_2_, and 100 mM ATP. For the phosphorylation of DivIVA and the DivIVA mutants by STK, the enzyme/substrate ratio was 1:20 with 0.5 mM kinase. The reaction was started with the addition of the kinase and conducted at 37°C for 30 min. The reaction was stopped by the addition of SDS-PAGE sample buffer, and 10 μL of the reaction mixture were subjected to electrophoresis. In each case, the reaction products were separated on a 12% SDS-polyacrylamide gel and transferred onto a polyvinylidene difluoride (PVDF) membrane. The proteins were detected with an anti-phosphothreonine polyclonal antibody or an anti-phosphoserine monoclonal antibody.

### Microscopy.

For transmission electron microscopy, cells were prepared as described previously (49). When the cells were grown to an OD_600_ of 0.5, 5 mL of bacterial culture was collected and centrifuged at 6,000 × *g* for 10 min at 4°C, and the cells were resuspended in normal saline. After being washed three times, the cells were resuspended with 2 mL of 2.5% glutaraldehyde and fixed overnight at 4°C. The samples were refixed in 1% osmic acid followed by acetone gradient dehydration, gradient mixed resin and acetone infiltration, embedding, ultrathin sectioning, and positive staining. Final samples were observed using an H-7650 transmission electron microscope (Hitachi, Japan).

For 3D-SIM microscopy and PG staining, HADA (blue) and TADA (orange) dissolved in dimethyl sulfoxide (0.2 M) were used to label nascent PG sites. Alexa Fluor 647 (AF 647; Thermo Scientific) was dissolved in dry acetonitrile (10 mg/mL) for cell membrane staining. After the OD_600_ value reached 0.5, the cells were transferred to 5 mL of TSB medium at 1:100, and 5 μL of HADA was added to the culture for 30 min to observe the synthesis of PG in one cycle. To measure the length of peripheral PG, the HADA-labeled cells in which the cell invagination has not started were selected, and the length of peripheral PG was measured for at least 100 cells using the ImageJ software. In order to further characterize the PG synthesis, two dyes were used successively to stain the cells. HADA was first used to stain the log-phase cells for 10 min and then chased with TADA for 30 min. The PG-labeled cells were centrifuged at 6,000 × *g* for 5 min, washed three times with PBS, mixed with 200 μL of AF647 with a final concentration of 0.25 mg/mL, and incubated at 37°C for half an hour. After three washes in PBS, imaging was performed with a structured illumination microscope (SIM) (Nikon Instruments, Inc., Tokyo, Japan).

Localization of fluorescently labeled proteins was performed as described previously (58) with some modifications. Cells expressing the GFP fusion protein were cultured in TSB medium at 37°C under the control of the inducible P_ATc_ promoter by adding ATc (anhydrotetracycline) to the TSB medium at a final concentration of 200 ng/mL and cultured for half an hour, and then 5 μL of HADA was added followed by incubation for 4 min. A total of 5 μL of the culture was spotted on microscope slides and covered with 1% agarose gel. An electron-multiplying charge-coupled device camera (iXon DU-897; Andor) and a total internal reflection fluorescence objective (100; numerical aperture [NA], 1.49; CFI Apo TIRF; Nikon Instruments, Inc.) were attached to a Nikon structured illumination microscope (N-SIM; Nikon Instruments, Inc.) to produce super-resolution images. Before direct reconstruction, the images were taken using the Nikon NIS-Elements AR program at various phases and angles of illumination.

### Coimmunoprecipitation and mass spectrometry.

Co-IP was performed using an anti-DivIVA antibody produced in mice. S. suis SC-19 and Δ*divIVA* strains were grown in TSB to an OD_600_ of 0.2 to 0.4. PBS buffer was used to resuspend the cells. A final concentration of 0.1% (vol/vol) paraformaldehyde solution was added for cross-linking at 37°C for 1 h. To stop the cross-linking process, 1.0 M glycine was added and incubated at 25°C for 10 min. The cells were then harvested, washed, and resuspended with the buffer containing 50 mM Tris-HCl (pH 7.4), 150 mM NaCl, 5 mg/mL lysozyme, 1% Triton X-100 (vol/vol), and 1% phenylmethylsulfonyl fluoride (vol/vol). The bacterial debris was eliminated after sonication by centrifugation at 14,000 × g for 10 min at 4°C. The total amount of protein of the cell lysate was normalized using a Micro BCA protein assay kit (Cwbiotech, Beijing, China). After removing nonspecific proteins from the cell lysate with 5 μL each of protein A and protein G, 5 μL of anti-DIvIVA serum was added to 500 μL of the cell lysate and mixed overnight at 4°C. Then, 5 μL of protein A and protein G were then added, incubated at 4°C for 2 h, and centrifuged at 12,000 × *g* for 1 min to obtain the precipitated antigen-antibody complex. The pellet was washed three times with wash buffer (50 mM Tris-HCl, 150 mM sodium chloride, 1% Nonidet P-40, and 0.05% sodium deoxycholate). The precipitant was tested by conventional immunoblotting to detect whether the target protein was pulled down, and the qualified precipitant was used to detect DivIVA-interacting proteins by mass spectrometry. Mass spectrometry analysis was performed using a Q Exactive Plus LC/MS system from Thermo. The mass spectral data generated by Q Exactive Plus were searched by MaxQuant (v1.6.2.10), and the database search algorithm used was MaxLFQ. The database used for the search was the Proteomic Reference Database of S. suis 2 in UniProt.

### Bacterial two-hybrid assay.

The BTH tests were carried out according to the manufacturer’s instructions (Euromedex). In the plasmids pUT18 and pKT25, the genes encoding our target proteins were cloned in-frame with either the T18- or T25-encoding gene. The plasmids were then transformed into E. coli BTH101. Three randomly selected colonies were chosen from the overnight incubation of the transformants, grown to the log phase, and then spotted (5 μL) onto LB agar plates containing ampicillin (100 mg/mL), kanamycin (50 mg/mL), IPTG (0.5 mM), and X-gal (40 mg/mL). After overnight incubation at 30°C, the results were documented. Quantitative analysis of protein interactions was achieved by measuring the activity of β-galactosidase as described previously (59). Briefly, the cells harboring the pUT18- and pKNT25-derived plasmids were incubated at 30°C for 16 h. The cells were washed and diluted with M63 culture medium, and the OD_600_ was measured. The cells were then lysed with SDS and chloroform. The cell lysate was mixed with PM2 medium followed by incubation at room temperature for 10 to 20 min. Then, 1 M Na_2_CO_3_ was added to terminate the reaction. The absorbance at 405 nm (OD_405_) was recorded using a microplate reader. The enzymatic activity is calculated as: 1,000 × (OD_405_ in sample well − OD_405_ in control wells)/OD_600_ in sample well – OD_600_ in control well/time (min) of incubation.

### Peptidoglycan hydrolysis assay.

Peptidoglycan was extracted from strain S. suis SC-19 as described previously (60, 61). The peptidoglycan hydrolysis assay was performed as previously described (39, 40). Briefly, the extracted peptidoglycan was incubated with 25 mM remazol brilliant blue (RBB) in 0.2 M NaOH at 37°C overnight. Then, the mixture was neutralized with HCl, and RBB-labeled peptidoglycan was pelleted by centrifugation and washed with water. The RBB-labeled PG was mixed with the indicated protein or mutanolysin (10 U) in PBS followed by incubation at 37°C. At each indicated time point, the reaction was terminated by incubating the samples at 95°C for 5 min. Afterward, the sample was subjected to centrifugation at 21,000 × *g* for 30 min at room temperature. The supernatant was collected, and the absorbance value at 595 nm was measured.

### Image analysis.

Cell length and width of strains grown in TSB medium were measured with ImageJ software from AF647 staining images taken by 3D-SIM microscopy from three independent experiments. The measurement of the length of the nascent peripheral PG was based on the 30-min HADA-stained photos taken by the 3D-SIM microscope. Only the nascent peripheral PG of those cells whose peripheral PG synthesis was completed and septal PG synthesis just started (as depicted in Fig. 3C) was counted. GraphPad Prism 8’s unpaired Student’s *t* test was used for statistical analysis.
