# Supplementary material for: DivIVA Interacts with the Cell Wall Hydrolase MltG To Regulate Peptidoglycan Synthesis in Streptococcus suis
Source: Microbiol Spectr. 2023 May 22;11(3):e04750-22. doi: 10.1128/spectrum.04750-22 (PMC10269899; doi:10.1128/spectrum.04750-22)
Supplement: Supplemental file 1 — Fig. S1. Download spectrum.04750-22-s0001.pdf, PDF file, 0.1 MB [file spectrum.04750-22-s0001.pdf]

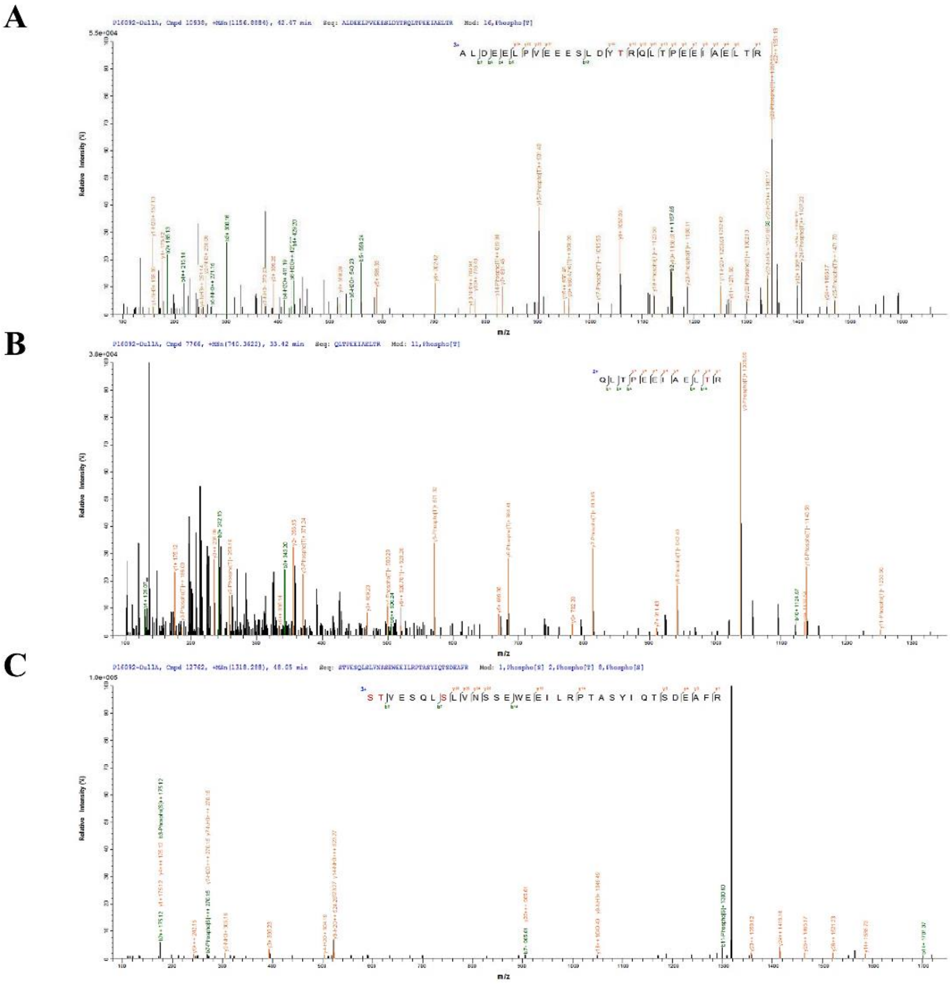

Figure S1. Identification of DivIVA phosphorylated sites. (A) Mass spectrum data showing that DivIVA is phosphorylated at Thr199. (B) Mass spectrum data showing that DivIVA is phosphorylated at Thr211. (C) Mass spectrum showing that DivIVA is phosphorylated at one of Ser145, T146, and S152.
