# Supplementary material for: DivIVA Interacts with the Cell Wall Hydrolase MltG To Regulate Peptidoglycan Synthesis in Streptococcus suis
Source: Microbiol Spectr. 2023 May 22;11(3):e04750-22. doi: 10.1128/spectrum.04750-22 (PMC10269899; doi:10.1128/spectrum.04750-22)
Supplement: Supplemental file 2 — Fig. S2. Download spectrum.04750-22-s0002.pdf, PDF file, 0.2 MB [file spectrum.04750-22-s0002.pdf]

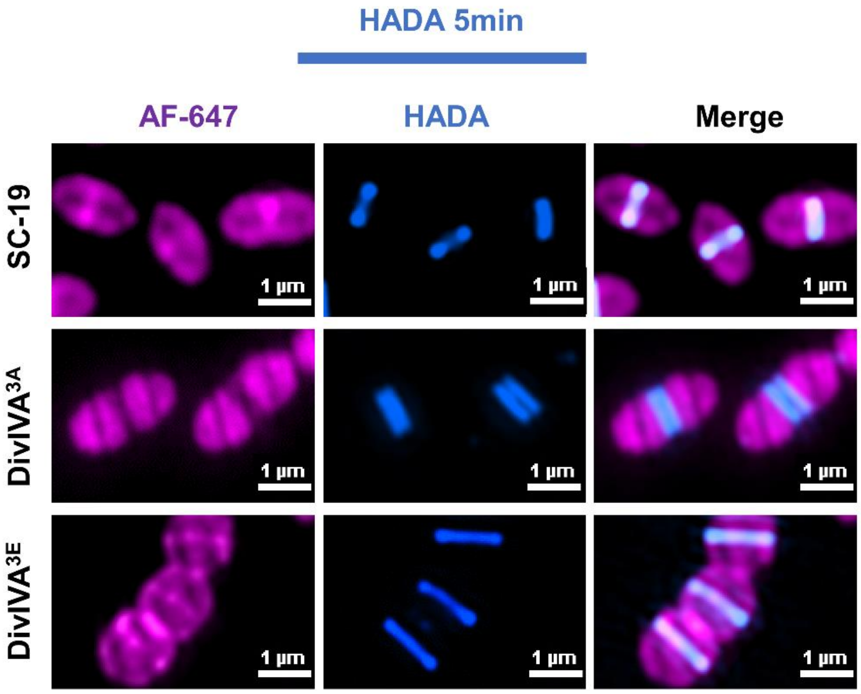

Figure S2. DivIVA phosphorylation does not affect the site of septal PG synthesis. The mid-log phase cells of *S. suis* SC-19, DivIVA^{3A}, and DivIVA^{3E} were pulsed with HADA dye for 5 min followed by staining with AF-647. The cells were washed and imaged using a structured illumination (SIM) microscope. The scale bar is 1μm.
