# Supplementary material for: DivIVA Interacts with the Cell Wall Hydrolase MltG To Regulate Peptidoglycan Synthesis in Streptococcus suis
Source: Microbiol Spectr. 2023 May 22;11(3):e04750-22. doi: 10.1128/spectrum.04750-22 (PMC10269899; doi:10.1128/spectrum.04750-22)
Supplement: Supplemental file 3 — Fig. S3. Download spectrum.04750-22-s0003.pdf, PDF file, 0.2 MB [file spectrum.04750-22-s0003.pdf]

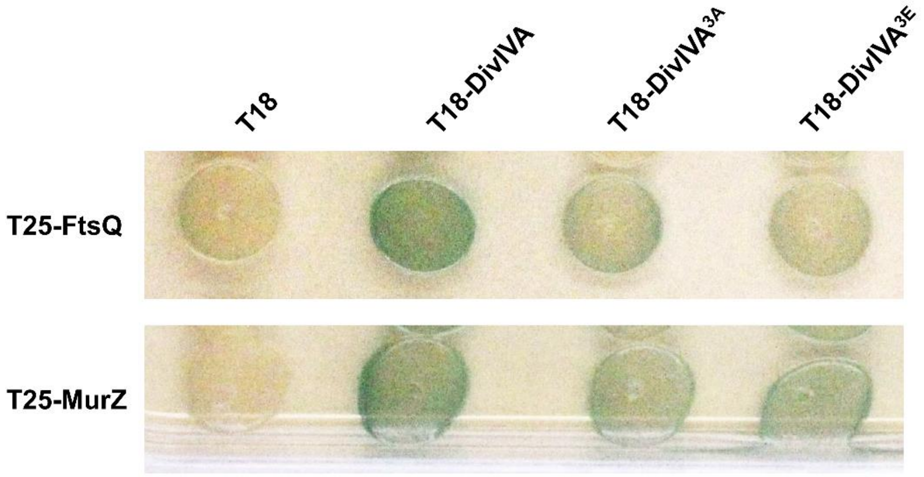

Figure S3. DivIVA phosphorylation does not affect the interaction with FtsQ and MurZ. *E. coli* BTH101 (Δ*cya*) carrying pUT18 or pKNT25-derived plasmids were grown to the mid-log phase and 5 μL spotted on LB agar plates containing X-gal, incubated at 30 ℃, and imaged. pUT18 was used as a negative control.
