# Supplementary material for: DivIVA Interacts with the Cell Wall Hydrolase MltG To Regulate Peptidoglycan Synthesis in Streptococcus suis
Source: Microbiol Spectr. 2023 May 22;11(3):e04750-22. doi: 10.1128/spectrum.04750-22 (PMC10269899; doi:10.1128/spectrum.04750-22)
Supplement: Supplemental file 4 — Fig. S4. Download spectrum.04750-22-s0004.pdf, PDF file, 0.2 MB [file spectrum.04750-22-s0004.pdf]

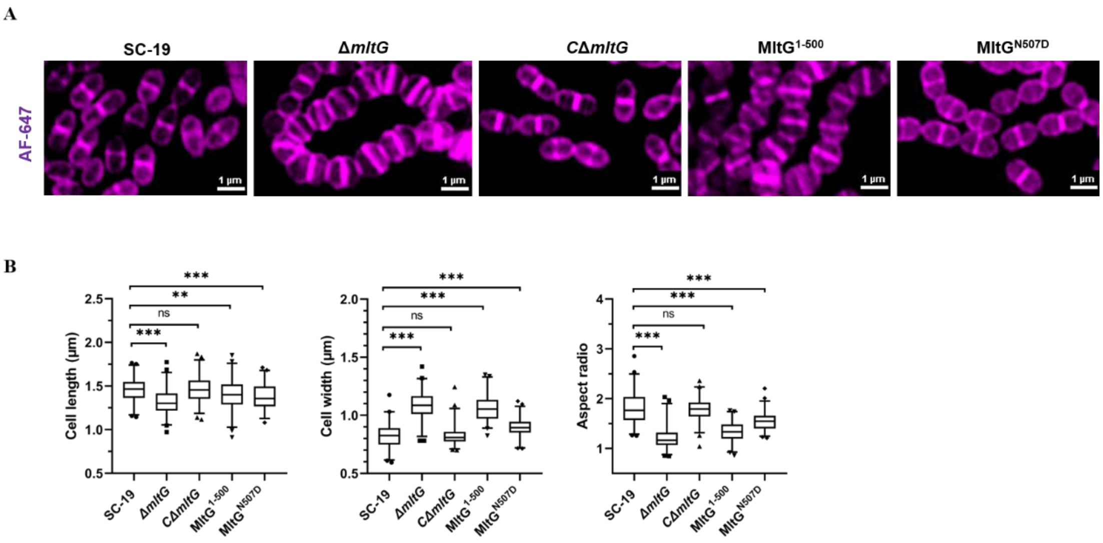

Figure S4. The enzyme activity of MltG is required for its function in regulating cell morphology of *S. suis*. (A) Morphology analysis. The cells of strain *S. suis* SC-19, Δ*mltG*, CΔ*mltG*, $MltG^{1\text{-}500}$and $MltG^{N507D}$ at the mid-log phase were stained with AF-647 dye followed by SIM analysis. The scale bar is 1μm. (B) Measurement of cell lengths, widths, and aspect ratios (cell length to width ratio). One hundred or more cells from two independent experiments were measured as described in Materials and Methods for each strain. P values were obtained by two-tailed, unpaired Student's *t* test. *** denotes P < 0.001. ** denotes P < 0.01. ns denotes no significance.
