# Supplementary material for: DivIVA Interacts with the Cell Wall Hydrolase MltG To Regulate Peptidoglycan Synthesis in Streptococcus suis
Source: Microbiol Spectr. 2023 May 22;11(3):e04750-22. doi: 10.1128/spectrum.04750-22 (PMC10269899; doi:10.1128/spectrum.04750-22)
Supplement: Supplemental file 5 — Table S1. Download spectrum.04750-22-s0005.doc, DOC file, 0.08 MB [file spectrum.04750-22-s0005.doc]

**Table S1** Primers used in this work

| **Primers** | **Sequence (5ʹ-3ʹ) *^a^*** | **target** |
| --- | --- | --- |
| **General PCR amplification** | | |
| **For pET28a-*divIVA*** |  |  |
| *divIVA*-F (*BamHI*) | AGCAAATGGGTCGCGGATCCATGGCACTTACAGCATTAGAATTAAAA | *divIVA* coding  sequence |
| *divIVA*-R (*EcoRI*) | TGTCGACGGAGCTCGAATTCTTATTCTTCTATTGAAAGTACTACAGATTCTCC |
| *divIVA*S145A-R | CTACTGTTGCTTTTAGACGCTGATG |
| *divIVA*S145A-F | TCTAAAAGCAACAGTAGAAAGCCAA |
| *divIVA*T146A-R | TTTCTACTGCTGATTTTAGACGCTG |
| *divIVA*T146A-F | AAAATCAGCAGTAGAAAGCCAATTA |
| *divIVA*S152A-R | TAACCAGTGCTAATTGGCTTTCTAC |
| *divIVA*S152A-F | CCAATTAGCACTGGTTAATTCATCT |
| *divIVA*T199A-R | ATTGACGTGCATAATCCAAACTTTC |
| *divIVA*T199A-F | GGATTATGCACGTCAATTGACACCA |
| *divIVA*T211A-R1 | TCTCAAAAGCTGCTGCCTGACGAGCTAATTCTGCAATCT |
| *divIVA*T211A-R2 | GAAAGTACTACAGATTCTCCACTCTCAAAAGCTGCTGCC |
| *divIVA*T211A-R3 | CCGCTCGAGTTATTCTTCTATTGAAAGTACTACAGATTC |
| **Homologous recombination** |  |  |
| **For pSET4s-A-B** |  |  |
| *divA*-F (*SmaI*) | CGACTCTAGAGGATCCCCGGGTGGCATACGCTTTGCTAAGTTG | Upstream fragment  of *divIVA* (1-1000  bps) |
| *divA*-R | CTTTGCTTATGATTTCTACTAAGTTTTAACAATGTCTAACTTTAATTTTATC |
| *divB-*F | GACATTGTTAAAACTTAGTAGAAATCATAAGCAAAGACAAGGTCG | Downstream fragment of *divIVA* (1-1000 bps) |
| *divB*-R (*EcoRI*) | AAAACGACGGCCAGTGAATTCGCCAAGACTTGCTCAATAGGAAGG |
| *mltG*A-F (*BamHI*) | AGGTCGACTCTAGAGGATCCAATCATTGCAACCATTGATGC | Upstream fragment  of *mltG* (1-1000  bps) |
| *mltG*A-R | CCACATAATTTTGTTTTAAGAATTTAATTCGTATCCTTTGTCACGCT |
| *mltG*B-F | AGCGTGACAAAGGATACGAATTAAATTCTTAAAACAAAATTATGTGG | Downstream fragment of *mltG* (1-1000 bps) |
| *mltG*B-R (*EcoRI*) | AAAACGACGGCCAGTGAATTCCCTGCTGGAGTGACTCTCTAC |
| **For pSET2-P*_divIVA_*-*divIVA*** |  |  |
| Cp*div*-F (*BamHI*) | AGGTCGACTCTAGAGGATCCTCAAATCAATTGCCTAGACCGAC | Promoter fragment of *ftsA* (1-461 bps) |
| Cp*div*-R | TAAGTGCCATCTTTCCTCCTCAAATACCTTTACTTTCTAACACCTTATT |
| C*div*-F | AAAGTAAAGGTATTTGAGGAGGAAAGATGGCACTTACAGCAT | *divIVA* coding sequence |
| C*div*-R (*EcoRI*) | AAACGACGGCCAGTGAATTCTTATTCTTCTATTGAAAGTACTACAGATTCTCC |
| **For pSET2-P*_mltG_*-*mltG***  CP*mltG*-F(*BamHI*)  CP*mltG*-R(*EcoRI*)  **For pSET2-P*_mltG_*-*mltG^1-500^***  CP*mltG^1-500^*-F(*BamHI*)  CP*mltG^1-500^*-R(*EcoRI*)  **For pSET2-P*_mltG_*-*mltG^N507D^***  507U-F(*BamHI*)  507U-R1  507U-R2  507D-F1  507D-F2  507D-R(*EcoRI*)  **For pSET4s-A-m*divIVA-*B** | AGGTCGACTCTAGAGGATCCCGGATGCCTATAAGGGACAGG  AAACGACGGCCAGTGAATTCTTACTCATTATTAAGATGTGCATTTACATACT  AGGTCGACTCTAGAGGATCCCGGATGCCTATAAGGGACAGG  AAACGACGGCCAGTGAATTCTTATGCATTCAAGCGGTTAAAGAAGA  AGGTCGACTCTAGAGGATCCCGGATGCCTATAAGGGACAGG  AGGCATTGCTGCATTCAAGC  ATTGCAATATCAGATTGTAGAGGCATTGCTGCATTCAAGC  CTTGTATGCTCAGGGTAAACTTGGT  CTACAATCTGATATTGCAATCTTGTATGCTCAGGGTAAACTTGGT  AAACGACGGCCAGTGAATTCTTACTCATTATTAAGATGTGCATTTACATACT |  |
| m*div*A-F(*SmaI*) | CGACTCTAGAGGATCCCCGGGTGGCATACGCTTTGCTAAGTTG | Upstream fragment  of *divIVA* (1-1000  bps) |
| m*div*A-R | GTGCCATCTTTCCTCCTAAGTTTTAACAATGTCTAACTTTAATTTTATC |
| m*div*-F | AACTTAGGAGGAAAGATGGCACTTACAGCATTAGAATTAAAAG | the *divIVA* sequence with phosphorylation site mutations |
| m*div*-R | TGCTTATGATTTCTATTATTCTTCTATTGAAAGTACTACAGATTCTCC |
| m*divB*-F | TCAATAGAAGAATAATAGAAATCATAAGCAAAGACAAGGTCG | Downstream fragment of *divIVA* (1-1000 bps) |
| m*divB*-R (*EcoRI*) | AAAACGACGGCCAGTGAATTCGCCAAGACTTGCTCAATAG |
| **For pSET2-P_ATc_-GFP-MltG** |  |  |
| pAtc-F (*BamHI*) | AGGTCGACTCTAGAGGATCCTTAAGACCCACTTTCACATTTAAGTTGT | ATc-inducible operon sequence |
| pAtc-R | TGCTGTAAGTGCCATTATATTACTCTCCTTTGAGTTTAAAATTGTTC |
| *gfp*-F | AAGGAGAGTAATATAATGGCACTTACAGCATTAGAATTAAAA | *gfp* coding sequence |
| *gfp*-R | CACTCCGGATCCCTCGAGTTTGTATAGTTCATCCATGCCATGT |
| *gmltg*-F | AAACTCGAGGGATCCGGAGTGACAAAGGATACGAATGAAAAAAA | *mltG* coding sequence |
| *gmltg*-R (*EcoRI*) | AAACGACGGCCAGTGAATTCTTACTCATTATTAAGATGTGCATTTACATACTT |
| **For Bacterial two hybrid** |  |  |
| pUT18*mltG*-F (*SalI*) | GGGCCCCCCCTCGAGGTCGACGGTGACAAAGGATACGAATGAAAAAAA | *mltG* coding sequence |
| pUT18*mltG*-R (*SalI*) | GCTTATCGATACCGTCGACCTCTCATTATTAAGATGTGCATTTACATACTTAG |
| pKT25*mltG*-F (*BamHI*) | AGGGTCGACTCTAGAGGATCCCGTGACAAAGGATACGAATGAAAAAAA | *mltG* coding sequence |
| pKT25*mltG*-R (*BamHI*) | TGGATAGGTACCCGGGGATCCTTACTCATTATTAAGATGTGCATTTACATACT |
| pUT18*div*-F (*SalI*) | GGGCCCCCCCTCGAGGTCGACGATGGCACTTACAGCATTAGAATTAAAA | *divIVA* coding sequence |
| pUT18*div*-R (*SalI*) | GCTTATCGATACCGTCGACCTTTATTCTTCTATTGAAAGTACTACAGATTCTCC |
| pKT25*div*-F (*BamHI*) | AGGGTCGACTCTAGAGGATCCCATGGCACTTACAGCATTAGAATTAAAA | *divIVA* coding sequence |
| pKT25*div*-R (*BamHI*) | TGGATAGGTACCCGGGGATCCTTCTTCTATTGAAAGTACTACAGATTCTCC | *divIVA* coding sequence |

***^a^*** Underlined sequences represent the restriction sites. The shadowed codons of DivIVA phosphorylation sites are replaced by the codon of alanine.
