# Supplementary material for: DivIVA Interacts with the Cell Wall Hydrolase MltG To Regulate Peptidoglycan Synthesis in Streptococcus suis
Source: Microbiol Spectr. 2023 May 22;11(3):e04750-22. doi: 10.1128/spectrum.04750-22 (PMC10269899; doi:10.1128/spectrum.04750-22)
Supplement: Supplemental file 6 — Table S2. Download spectrum.04750-22-s0006.doc, DOC file, 0.10 MB [file spectrum.04750-22-s0006.doc]

**Table S2. Bacterial strains and plasmids used in this study**

| **Strain/plasmid** | **Characteristics*^a^*** | **Source** |
| --- | --- | --- |
| **Strains** |  |  |
| *S. suis* strains |  |  |
| SC-19 | A virulent *S. suis* serotype 2 strain isolated from diseased pig of the China’s outbreak in 2005, the wild-type |  |
| Δ*divIVA* | The *divIVA* deletion mutant of strain SC-19 | This work |
| Δ*mltG* | The *mltG* deletion mutant of strain SC-19 | This work |
| CΔ*divIVA* | SC-19, Δ*divIVA* *divIVA*^+^; The complemented strain of Δ*divIVA*; Spc^r^ | This work |
| CΔ*mltG* | SC-19, Δ*mltG* *mltG*^+^; The complemented strain of Δ*divIVA*; Spc^r^ | This work |
| DivIVA^3A^ | SC-19, Δ*divIVA* blank:: DivIVA^S145A-T199A-T211A^ | This work |
| DivIVA^3E^ | SC-19, Δ*divIVA* blank:: DivIVA^S145E-T199E-T211E^ | This work |
| MltG^1-500^ | SC-19, Δ*mltG* *mltG^1-500^*; Spc^r^ | This work |
| MltG^N507D^ | SC-19, Δ*mltG* *mltG^N507D^*; Spc^r^ |  |
| SC19-GMltG | SC-19, pSET2-P*_ATc_-gfp-mltG* | This work |
| Δ*divIVA*-GMltG | SC-19, Δ*divIVA,* pSET2-P*_ATc_-gfp-mltG* | This work |
| DivIVA^3A^-GMltG | SC-19, DivIVA^3A^, pSET2-P*_ATc_-gfp-mltG* | This work |
| DivIVA^3E^-GMltG | SC-19, DivIVA^3E^, pSET2-P*_ATc_-gfp-mltG* | This work |
| *E. coli* strains |  |  |
| DH5α | Cloning host for maintaining recombinant plasmids | Trans |
| BL21 (DE3) | Expression host for exogenous protein production | Trans |
| BTH101 | Host Strain for pUT18 and pKNT25 recombinants; Str^r^ | Stratagene |
| **Plasmids** |  |  |
| pUT18 | Plasmid used in BACTH analysis; Amp^r^ | Stratagene |
| pKNT25 | Plasmid used in BACTH analysis; Chl^r^ | Stratagene |
| pT18-*divIVA* | T18 domain fused to DivIVA; Amp^r^ | This work |
| pT18-*mltG* | T18 domain fused to MltG; Amp^r^ | This work |
| pT18-*zip* | T18 domain fused to Zip; positive control; Amp^r^ | Stratagene |
| PT25-*divIVA* | T25 domain fused to DivIVA; Chl^r r^ | This work |
| PT25-*mltG* | T25 domain fused to MltG; Chl^r^ | This work |
| PT25-*zip* | T25 domain fused to Zip; positive control; Chl^r^ | This work |
| pET-28a | His tag fusion expression vectors; Kan^r^ | Novagen |
| pET-STK | pET-28a with the full-length STK; Kan^r^ |  |
| pET-N-STK | pET-28a with the full-length truncated N-STK; Kan^r^ |  |
| pET-DivIVA | pET-28a with the full-length DivIVA; Kan^r^ | This work |
| pET-DivIVA^S145A^ | pET-28a with S145A site mutant DivIVA; Kan^r^ | This work |
| pET- DivIVA^S146A^ | pET-28a with S146A site mutant DivIVA; Kan^r^ | This work |
| pET- DivIVA^T199A^ | pET-28a with T199A site mutant DivIVA; Kan^r^ | This work |
| pET- DivIVA^T211A^ | pET-28a with T211A site mutant DivIVA; Kan^r^ | This work |
| pET- DivIVA^2TA^ | pET-28a with the T199A/T211A mutant DivIVA; Kan^r^ | This work |
| pSET2 | The *E. coli* DH5α-*S. suis* shuttle vector; Spc^r^ |  |
| P*_divIVA_*-*divIVA* | pSET2 with the expression cassette of *divIVA*; Spc^r^ | This work |
| pSET2-*mltG* | pSET2 with *mltG* gene containing its promoter; Spc^r^ | This work |
| pSET2-*mltG*^1-500^ | pSET2 with *mltG^1-500^* containing its promoter; Spc^r^ | This work |
| pSET2-*mltG*^N507D^ | pSET2 with *mltG*^N507D^ containing its promoter; Spc^r^ | This work |
| pSET4s | Thermosensitive suicide vectors for gene replacement  in *S. suis*; Spc^r^ |  |
| pSET4s-AB-*divIVA* | For the deletion of *divIVA* in *S. suis*; Spc^r^ | This work |
| pSET4s-AB-*mltG* | For the deletion of *mltG* in *S. suis*; Spc^r^ | This work |
| pSET4s-AB-DivIVA^3A^ | To construct the dephosphorylated mutant; Spc | This work |
| pSET4s-AB-DivIVA^3E^ | To construct the phosphorylation-mimicking mutant; Spc | This work |
| P*_divIVA_*-*divIVA* | pSET2 with the expression cassette of *divIVA*; Spc^r^ | This work |
| pMIDG310 | A plasmid containing the GFP coding sequence; Spc^r^ |  |
| pSSTete2-*lacZ* | A plasmid containing ATc inducible promoter; Spc^r^ |  |
| P_ATc_-*gfp*-*mltG* | pSET2 with the inducible expression cassette of GFP-MltG | This work |

***^a^***Str^r^, streptomycin resistant; Spc^r^, spectinomycin resistant; Kan^r^, kanamycin resistant; Chl^r^, chloramphenicol resistant; Tet^r^, tetracycline resistant; Amp^r^, ampicillin resistant.

**References**

1. Zhang A, Xie C, Chen H, Jin M. 2008. Identification of immunogenic cell wall-associated proteins of Streptococcus suis serotype 2. Proteomics 8:3506-15.

2. Zhang C, Sun W, Tan M, Dong M, Liu W, Gao T, Li L, Xu Z, Zhou R. 2017. The Eukaryote-Like Serine/Threonine Kinase STK Regulates the Growth and Metabolism of Zoonotic Streptococcus suis. Front Cell Infect Microbiol 7:66.

3. Takamatsu D, Osaki M, Sekizaki T. 2001. Construction and characterization of Streptococcus suis-Escherichia coli shuttle cloning vectors. Plasmid 45:101-13.

4. Takamatsu D, Osaki M, Sekizaki T. 2001. Thermosensitive suicide vectors for gene replacement in Streptococcus suis. Plasmid 46:140-8.

5. Zhang T, Ding Y, Li T, Wan Y, Li W, Chen H, Zhou R. 2012. A Fur-like protein PerR regulates two oxidative stress response related operons dpr and metQIN in Streptococcus suis. BMC Microbiol 12:85.

6. Zhang L, Zou W, Ni M, Hu Q, Zhao L, Liao X, Huang Q, Zhou R. 2022. Development and Application of Two Inducible Expression Systems for Streptococcus suis. Microbiol Spectr 10:e00363-22.
